# Use of Social Media for Cancer Prevention Through Neighborhood Social Cohesion: Protocol for a Feasibility Study

**DOI:** 10.2196/28147

**Published:** 2021-07-30

**Authors:** Ingrid Oakley-Girvan, Jessica L Watterson, Cheryl Jones, Lauren C Houghton, Marley P Gibbons, Kajal Gokal, Kate Magsamen-Conrad

**Affiliations:** 1 Medable Palo Alto, CA United States; 2 The Data and Technology Proving Ground Program The Public Health Institute Oakland, CA United States; 3 Jeffrey Cheah School of Medicine and Health Sciences Monash University Malaysia Selangor Malaysia; 4 Center for Healthcare Organizational and Innovation Research School of Public Health University of California Berkeley Berkeley, CA United States; 5 Manchester Centre for Health Economics The University of Manchester Manchester United Kingdom; 6 Department of Epidemiology Mailman School of Public Health Columbia University New York, NY United States; 7 Herbert Irving Comprehensive Cancer Center New York, CA United States; 8 School of Sport, Exercise, and Health Sciences National Centre for Sport and Exercise Medicine Loughborough University Loughborough United Kingdom; 9 Department of Communication Studies The University of Iowa Iowa City, IA United States

**Keywords:** social cohesion, mothers, neighborhood, physical activity, social media, social, behavior, health outcomes, socioeconomic status, community health, chronic disease, social network, feasibility, wellbeing, cancer

## Abstract

**Background:**

Social cohesion is associated with healthier behaviors and better health outcomes, and therefore may offer a mechanism for promoting better health. Low socioeconomic status (SES) communities face higher rates of chronic disease due to both community- and individual-level factors.

**Objective:**

The aim of this study is to leverage social cohesion to promote healthier behaviors and prevent chronic disease in a low SES community. This protocol outlines the methodology for a pilot study to assess the feasibility of an intervention (Free Time For Wellness [FT4W]) using a social networking platform (Nextdoor) with mothers living in an urban, low-income community to improve social cohesion and promote healthy behaviors.

**Methods:**

The study will involve three phases: (I) co-designing the intervention with mothers in the neighborhoods of interest, (II) implementing the intervention with community leaders through the social networking platform, and (III) evaluating the intervention’s feasibility. Phase I of the study will include qualitative data collection and analysis from in-depth, semistructured interviews and a co-design group session with mothers. Phases II and III of the study include a pre- and postintervention survey of participating mothers. Neighborhood-level data on social cohesion will also be collected to enable comparison of outcomes between neighborhoods with higher and lower baseline social cohesion.

**Results:**

As of March 2021, recruitment and data collection for this study are complete. This protocol outlines our original study plan, although the final enrollment numbers and intervention implementation deviated from our initial planned methodology that is outlined in this protocol. These implementation learnings will be shared in subsequent publications of our study results.

**Conclusions:**

Ultimately, this study aims to: (1) determine the barriers and facilitators to finding free time for wellness among a population of low-income mothers to inform the co-design process, and (2) implement and study the feasibility of an intervention that leverages social cohesion to promote physical activity in a community of low-income mothers. The results of this study will provide preliminary feasibility evidence to inform a larger effectiveness trial, and will further our understanding of how social cohesion might influence well-being.

**International Registered Report Identifier (IRRID):**

RR1-10.2196/28147

## Introduction

This protocol outlines the methodology for a pilot study to assess the feasibility of an intervention (Free Time For Wellness [FT4W]) using a social networking platform (Nextdoor) with mothers living in an urban, low-income community to improve social cohesion and to promote healthy behaviors.

Social cohesion describes the extent of connectedness and solidarity with groups [1], such as feelings of trust and inclusion in social settings [2]. Measures of social cohesion assess the degree to which individuals experience trusting relationships, cooperation, and participation in their communities [3]. High or average social cohesion is positively related to good self-rated health [2,3]. Social cohesion has also been found to be correlated with healthier behaviors and better health outcomes such as higher rates of physical activity [4]; lower rates of smoking, drinking, and depression [5]; lower BMI [6]; and lower rates of myocardial infarction [7]. Living in a cohesive community may improve health through the diffusion of health information and resources that enable individuals’ engagement in healthy behaviors [3,8]. Social cohesion may also impact neighborhood safety [9] or social norms that reduce risky behavior and increase mutual respect, thereby reducing stress [3].

Given the linkages between social cohesion and health outcomes, our study aims to build social cohesion to promote healthy behavior using the social media app Nextdoor. As the prevalence of social media has risen in our society, its use has been studied for health issues such as tobacco use, diet, physical activity, and sexual practices [10]. A recent study of a social media–based pilot intervention for weight loss among adults with low socioeconomic status (SES) was found to be feasible, with results demonstrating high rates of engagement, increases in social support, and decreases in body weight among participants [11]. In addition, a recent meta-analysis of 22 studies of social media interventions for weight loss or related behaviors found a modest but statistically significant weight loss effect of 1 kilogram [12]. Facebook is the most commonly used tool for similar social media lifestyle intervention research [13]; therefore, this study will add to this body of literature by designing and evaluating an intervention using a newer social media tool (Nextdoor) that prioritizes connections between people in the same geographic area through address verification.

This study will focus on co-designing and implementing the intervention in the urban, low-income community of Washington Heights, New York City (NYC). Communities of low SES are at increased risk of developing conditions such as cancer, heart disease, diabetes, and other chronic diseases [3,14,15]. This increased risk is due to factors at many levels, including the environment, community, family, and individual [16]. At the community level, neighborhoods perceived as unsafe, hostile, isolating socially or culturally, or that are extremely polluted have been linked to poor self-rated health and higher mortality [17,18]. Greater levels of stress [19] and poorer sleep quality [20] occur among people who perceive their neighborhoods as unsafe and esthetically unpleasing. High levels of reported stress are also related to being less likely to engage in healthy behaviors [21], and the Centers for Disease Control and Prevention reported that individuals of low SES may also be more likely to smoke cigarettes, to be obese, to develop diabetes, and to experience preventable hospitalizations [22]. Therefore, this study will focus on a low SES community where the risk of chronic disease is higher and there is greater potential to make an impact on these health disparities.

Finally, this study will focus on co-designing and implementing the intervention with mothers, given their ability to influence the health behaviors of their households. According to Yuma-Guerrero and colleagues [23], social cohesion may improve mothers’ engagement in physical activity, and thus directly impact individuals in that family because mothers make decisions that affect family health and model behavior for their children. Accumulating evidence [24] suggests that, ideally, cultivating risk-reduction behaviors begins in childhood, modeled by trusted caregivers and communities. We also selected mothers as the target population as they are more likely to have common interests and experiences that could facilitate building social cohesion, as opposed to a more heterogenous group. Therefore, this study will focus on cultivating social cohesion to promote healthy behaviors among mothers living in an urban, low-income community.

We hypothesize that an intervention using neighborhood-level social media to encourage behavioral activation and accountability to others will help to increase healthy behaviors among mothers living in an urban, low-income community.

## Methods

### Purpose

The purpose of this study is to co-design and assess the feasibility of the FT4W intervention. FT4W uses a social networking platform, Nextdoor, to improve social cohesion among mothers in a low-income, urban community (Washington Heights, NYC), with the ultimate goal of improving health behaviors that prevent chronic disease, such as physical activity. By focusing on building social cohesion, rather than explicitly discussing disease prevention or healthy behaviors, this intervention will employ a “stealth approach” to health promotion and will focus on addressing process motivation rather than on an explicit health outcome [25]. To our knowledge, no interventions using this approach to promote healthy behavior while building social cohesion have been performed in this target population with this social media platform.

This pilot study has two aims. Aim 1 is to identify the barriers and facilitators to free time for wellness activities, and to co-design an intervention with mothers from an urban, low-income community. Aim 2 is to assess the feasibility of the intervention, leveraging social cohesion to promote healthy behaviors using Nextdoor among mothers in Washington Heights.

### Premise of the Study

Although the exact intervention activities will be designed together with mothers from the target community, the research team has developed initial ideas for the intervention based on evidence, theory, and personal experience, and will seek feedback from mothers on these ideas. These initial ideas relate to the communication mode, program leadership, and intervention activities.

### Communication Mode: Nextdoor

In our initial conceptualization, mothers would enroll in a Nextdoor group with others from their neighborhood. As outlined above, social media interventions show promise for improving social support and healthier behaviors, and Nextdoor was selected as the platform of interest by the research team because of its focus on physical proximity and safety through address verification. The relevant features of Nextdoor that will be considered for utilization in this intervention are: (i) connecting participants to others living in their neighborhood, (ii) creating a group of study participants where plans for group wellness activities in their neighborhood (and other topics of interest) can be discussed among participants, and (iii) group moderators can post information or reminders about upcoming events or activities. It is anticipated that the Nextdoor platform will be used for communication, whereas the wellness activities themselves will take place in person in the participants’ neighborhood.

### Program Leadership: Community Champion

We plan to engage a community champion to promote enrollment and participation in the intervention. We plan that their role will include sharing information about activities with the group, facilitating conversations among group members in the online platform, participating in activities, and generally promoting interest and engagement. The idea to utilize a community champion to facilitate the intervention stems from Rogers' diffusion of innovation theory, and subsequent evidence that suggests champions may be able to best influence others to enroll and maintain engagement in the program [26,27]. We plan to identify interested community champions during the activities of Aim 1 (interviews and workshop) and to offer a stipend as a gesture of appreciation for their time.

### Intervention Activities: Alternating Childcare and Exercise

A potential intervention activity could include taking turns to watch children in a small group while other mothers go for a group walk, and then alternating roles so that all mothers have the opportunity to be active. This idea was inspired by personal experiences of the researchers, who had used Nextdoor in their own neighborhoods to arrange their own childcare and fitness activities.

Although these ideas are included here to illustrate the potential of this intervention, obtaining input from mothers during the co-design workshop is essential to ensure that the intervention fits their needs, and that they will be interested and motivated to participate.

**Figure 1:**
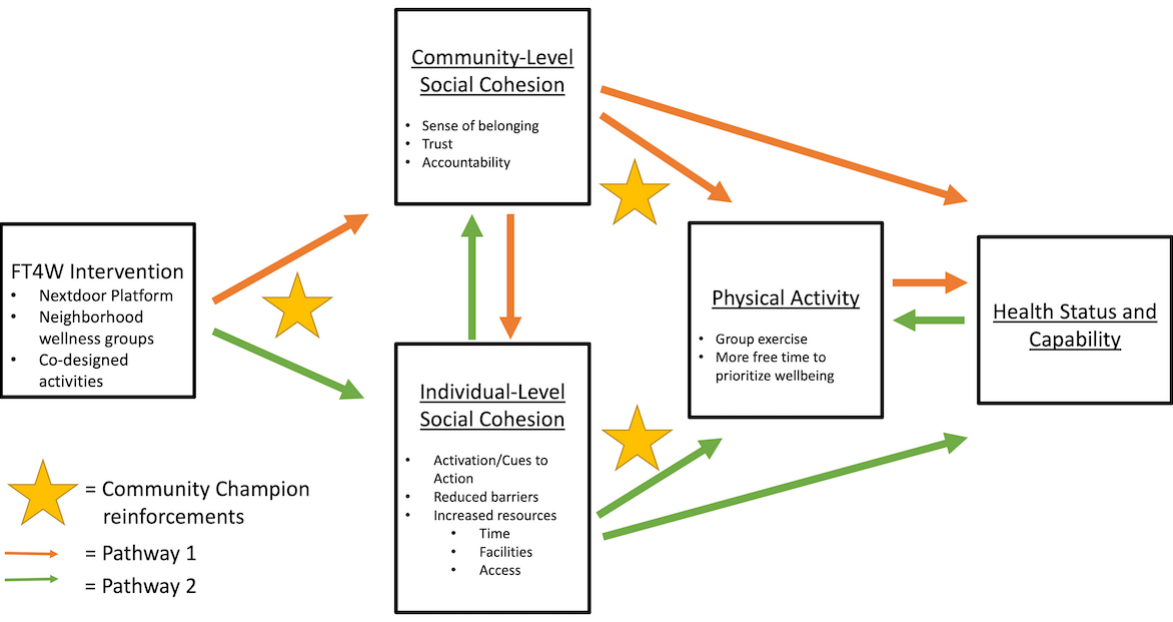
Hypothesized framework of behavior change. FT4W: Free Time For Wellness.

We hypothesize that the co-design FT4W intervention can lead to behavior change through the framework outlined in Figure 1. Specifically, we hypothesize that the co-designed FT4W intervention will promote social cohesion, both at a community and individual level. A community champion will be used to facilitate the Nextdoor group intervention and to reinforce positive group dynamics. We expect that increases in social cohesion will result in greater community-level trust and accountability, as well as individual-level resources and activation (or cues to action). In turn, mothers will adopt healthier behaviors such as higher levels of physical activity, and will experience better health and greater capability.

### Approach

#### Aim 1

To achieve Aim 1, the researchers will perform qualitative interviews and a co-design workshop with mothers from the low-income, urban community of Washington Heights, NYC.

First, semistructured, open-ended interviews will be performed with mothers to understand the barriers and facilitators to free time for wellness. These interviews will also explore mothers’ ability and willingness to use the social media platform Nextdoor. Approximately 12 mothers will be recruited for interviews from the Washington Heights community, although the final number will depend on when data saturation is met. Data saturation will be defined as “the degree to which new data repeat what was expressed in the previous data” [28]. In practice, this means that when the interviewers begin to hear the same comments being repeated in interviews, we will stop collecting new data and will begin analysis [28]. Recruiting will be performed through community groups, newsletters, Facebook groups, and listservs for mothers in the Washington Heights neighborhood, resulting in a convenience sample. The interviews will be recorded and transcribed, and the data will be inductively coded following grounded theory [29].

Second, a co-design workshop will be held with mothers in Washington Heights to design the content of the intervention. The workshop will include rotating, small group discussions at four “stations” to elicit ideas and feedback from mothers on four topics: (i) use of the Nextdoor platform and other communication technologies, (ii) ways to create additional free time in their schedules for wellness activities, (iii) the types of wellness activities they would like to participate in, and (iv) the characteristics they would like the community leader/facilitator to have. These topics were prioritized by the researchers as the main areas where feedback and input from participants were needed. The format of rotating small group discussions was chosen to facilitate easier conversation, where all participants would have the chance to speak and to give input on multiple topics.

A total of 20 mothers will be recruited to participate in the co-design workshop, starting with extending invitations to the mothers who participated in the interviews and asking them to invite other mothers from their community as well. In addition to this snowball sampling, flyers will be posted in the neighborhood to recruit additional mothers. Mothers’ input will be audio-recorded and collected through notes taken by the research team during the workshop. Analysis of these qualitative data will be performed immediately following the workshop by reviewing the audio recordings and notes as a research team, discussing common themes emerging from the results, and deciding on the final components of the intervention through consensus. Using the design input from the mothers at the co-design session, the research team will decide on: (i) *what* the intervention wellness activities will be, (ii) *how* intervention group communication will take place, (iii) *who* will facilitate group communication and wellness activities, (iv) *where* the wellness activities will take place, and (v) *when* the wellness activities will take place (frequency).

#### Aim 2

To achieve Aim 2, the research team will aim to enroll 30 mothers in the intervention group and will administer a baseline survey before the intervention begins and a follow-up survey after the intervention ends (4 months later). Mothers who attend the co-design workshop will be asked if they would like to participate in the intervention, and snowball sampling and neighborhood flyers will be used to recruit additional mothers until the target number is reached.

**Figure 2:**
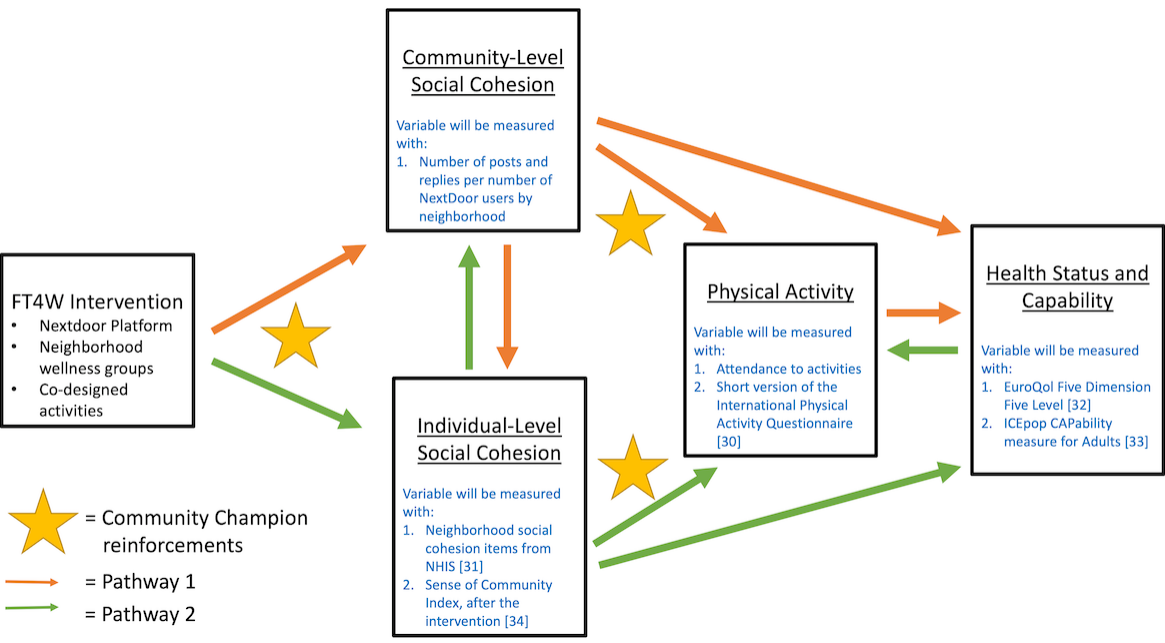
Hypothesized framework of behavior change with details on how each variable will be measured in Aim 2. NHIS: National Health Interview Survey; FT4W: Free Time For Wellness.

The baseline and follow-up survey will measure: physical activity (using the short version of the International Physical Activity Questionnaire [30]), individual-level perceptions of neighborhood social cohesion (using 4 questions from the National Health Interview Survey [31]), health status (using the EuroQol Five Dimension Five Level [32]), and capability (using the ICEpop CAPability measure for Adults [33]). The follow-up survey will also measure acceptability of the FT4W intervention (using questions to measure mothers’ perceptions on whether the intervention is effective at freeing up their time to participate in wellness activities), perceptions of community among FT4W participants (through the Sense of Community Index [34]), and self-reported attendance to activities. In addition to the survey, data will also be collected on study retention (through attendance to intervention activities and participation in group communications), community-level interactions on Nextdoor (number of posts and replies among all Nextdoor members in Washington Heights neighborhoods during the study period), and the cost of the intervention. The relationships of these measured variables to our hypothesized framework of behavior change are outlined in Figure 2, showing how each variable will be measured through the Aim 2 data collection.

A descriptive analysis will be performed for each quantitative outcome measure described above. Given the small sample size, it will not be possible to make statistical inferences of the effectiveness of the intervention; however, the instruments used to collect the data will be assessed for their sensitivity and relevance for use in a future larger-scale trial. We will also perform an economic cost-benefit analysis because the introduction of a new intervention requires efficient resource allocation to achieve the greatest outcome. Economic evaluation is a tool that is used to compare the costs and benefits of two or more interventions [35]. To fully assess the cost and benefits of a new intervention, a trial with a sufficiently large sample size is required. However, it is important to consider the design of a cost-benefit (effectiveness) analysis from the inception of a new intervention. The purpose of the economic analysis for this study will be to test data collection methods (eg, baseline and follow-up surveys) to ascertain the most relevant and sensitive outcome measures that can be used to capture changes. Cost data will be estimated by collecting costs for services that would need to be funded for the intervention to succeed/function, including the cost of renting a community space for an intervention activity.

## Results

As of March 2021, recruitment and data collection for this study is complete. This protocol outlines our original study plan, although final enrollment numbers and intervention implementation deviated from our initial planned methodology that is outlined in this protocol. These implementation learnings will be shared in subsequent publications of our study results.

## Discussion

This protocol outlines the study methodology for a multilevel participatory community intervention to promote healthier behaviors among mothers in a low-income neighborhood. Unhealthy lifestyles and low rates of physical activity are potentially modifiable risk factors for many chronic diseases. Low SES communities are at especially elevated risk for developing chronic diseases, and they experience many barriers to better health. Low-cost, scalable programs that could be implemented regardless of geography, but tailored to the needs of neighborhoods, could result in a significant positive impact on national and potentially international health outcomes.

This study involves a multidisciplinary research team with expertise in behavioral science and interventions, implementation science, health education, digital health, epidemiology, anthropology, psychology, health economics, and health communication. This study will also employ a multilevel approach to address barriers to healthy behavior at both the community and individual levels. Further, this study employs co-design methodology to engage the target community in the planning of the intervention, to ensure it meets their needs and is desirable and engaging. Finally, we have cultivated an informal partnership with the social media platform Nextdoor. Similarly, we plan to engage with community services in Washington Heights to ensure that the intervention is local and accessible for mothers in the community.

The proposed multilevel mixed methods study will harness neighborhood-based social networking to improve social cohesion and ultimately chronic disease prevention through enhanced healthy behaviors among mothers in Washington Heights. If successful, this work could help reduce persistent disparities in chronic disease incidence and outcomes among communities with low SES.

## Abbreviations

FT4W: Free Time for Wellness
NYC: New York City
SES: socioeconomic status
